# Femtosecond Laser-Pulse-Induced Surface Cleavage of Zinc Oxide Substrate

**DOI:** 10.3390/mi12060596

**Published:** 2021-05-21

**Authors:** Xi Yu, Fumihiro Itoigawa, Shingo Ono

**Affiliations:** 1 Department of Physical Science and Engineering, Nagoya Institute of Technology, Nagoya 466-8555, Japan; ono.shingo@nitech.ac.jp; 2 Department of Electrical and Mechanical Engineering, Nagoya Institute of Technology, Nagoya 466-8555, Japan; itoigawa.fumihiro@nitech.ac.jp

**Keywords:** femtosecond laser processing, laser-induced surface cleavage, multi-photon absorption

## Abstract

The induction of surface cleavage along the crystalline structure of a zinc oxide substrate (plane orientation: 0001) by femtosecond laser pulses (wavelength: 1030 nm) has been reported; a scanning electron microscope image of the one-pulse (pulse energy: 6–60 μJ) irradiated surface shows very clear marks from broken hexagons. This cleavage process differs from the general laser-induced melt process observed on the surfaces of narrower-bandgap semiconductors and other metal materials. This phenomenon is discussed using a multi-photon absorption model, and the pulse-energy dependence of the cleavage depth (less than 3 μm) is quantitatively analyzed. Laser-induced cleavage is found not to occur under multi-pulse irradiation; when more than four pulses are irradiated upon the same spot, the general laser-induced melt process becomes dominant. This cleavage–melt shift is considered to be caused by the enhancement of absorption due to the initial pulses, which is supported by our measurement of cathodoluminescence.

## 1. Introduction

Zinc oxide (ZnO) has been actively researched for a broad range of applications in the ultraviolet (UV) and visible optical regions [1,2]. Benefiting from a wide bandgap (~3.3 eV) and various defect levels, it has been studied for more than two decades and has applications both scientific (e.g., UV–visible emitters and detectors [3,4]) and daily life (e.g., sunscreen and display [5,6,7]). Beyond the UV–visible region, ZnO is reported to have high transmittance and a high refractive index in the terahertz (10^11^–10^13^ Hz) region [8,9]. The emission of terahertz waves from ZnO-based devices is very strong evidence that ZnO also has potential uses for terahertz components [8,10]. In both the UV–visible and terahertz regions, laser processing is an efficient method for realizing thin-film-based devices or nano/microstructure-based functional surfaces [11,12,13,14,15]. ZnO-based thin films fabricated by pulsed laser deposition have been widely reported by a vast number of researchers [2]; in most of these reports, a UV laser with a nanosecond order pulse duration was used. By contrast, ZnO-based functional surface structures fabricated by direct laser irradiation upon a ZnO substrate occur seldom, despite the appearance of cheaper lasers with extremely high outputs [16,17]. Recently, femtosecond laser-induced nano-ripples on ZnO surfaces have been separately reported by Hang et al. [18] and Liu et al. [19]. Multiple laser pulses are irradiated upon the ZnO substrate surface to fabricate structures with a size less than the wavelength of an incident laser pulse. Meanwhile, the single-pulse irradiation physics are not mentioned in such literature.

**Figure 1:**
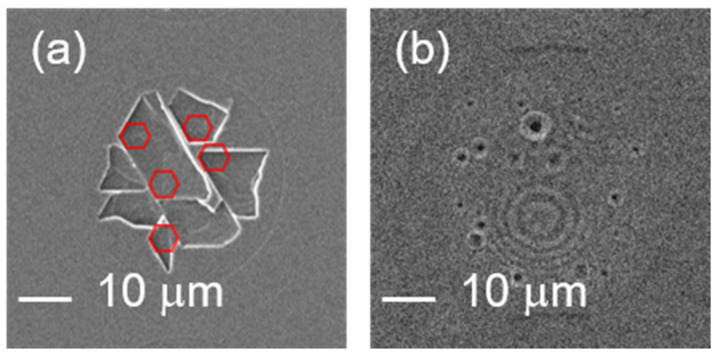
The one-pulse irradiated surface of ZnO (**a**) and Si (**b**) by a femtosecond laser with a wavelength of 1030 nm and pulse energy of 30 μJ. Corners with 120° angle randomly distributed in the one-pulse irradiated area are observed, which may be pieces from broken hexagon patterns.

Figure 1a shows the one-pulse irradiated ZnO substrate surface of our work; this is a very interesting morphology that differs from the laser-irradiated surface of the silicon (Si) substrate or some other metal materials [20,21,22]. As shown in Figure 1b, in general, these one-pulse irradiated marks on Si or other metal surfaces are considered to be caused by melting with heat. On the contrary, no melting was observed from the one-pulse irradiated spot of the ZnO substrate; instead, corners with a 120° angle (pieces from broken hexagon patterns) are observed.

To study these marvelous cleavage phenomena in detail, we performed one-pulse irradiation (pulse energy: 6–60 μJ) on the surface of the ZnO substrate, as well as multi-pulse (1–11 pulses) irradiation with the same pulse energy of 30 μJ. The dependence of pulse energy upon cleavage depth was discussed and quantitatively analyzed with a three-photon absorption model; a cleavage–melt shift was also observed after four laser pulses. This shift is attributed to the enhancement of absorption due to the initial pulses, and the mechanism is supported by our measurement of cathodoluminescence.

## 2. Experimental Details

**Figure 2:**
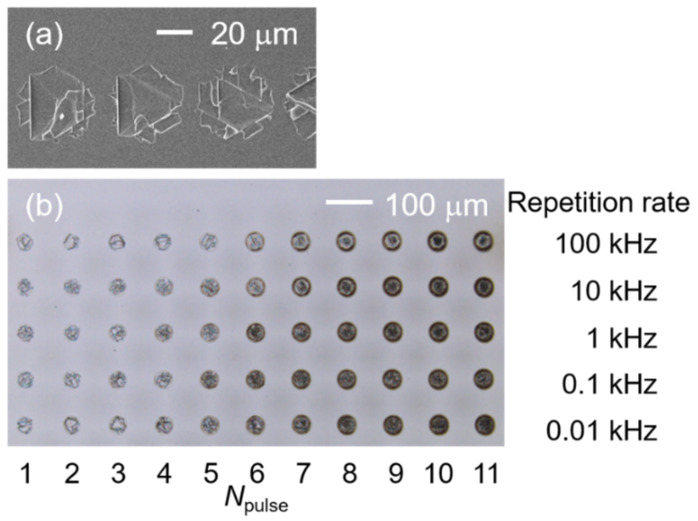
(**a**) SEM image of the one-pulse irradiated ZnO substrate surface under pulse energy of 60 μJ by scanning a laser beam in a line. (**b**) CLSM images of spots irradiated by laser pulses with different pulse numbers (*N*_pulse_) and repetition rates.

Femtosecond laser pulses from an Yb:KGW laser (central wavelength: 1030 nm; pulse duration: 700 fs; PHAROS PH1-10, Light Conversion, Vilnius, Lithuania) were irradiated and focused upon the surface of a ZnO substrate (orientation: 0001; hexagonal structure; purchased from CrysTec GmbH, Berlin, Germany) by an F-Theta lens (focus length: 200 mm; SILL 297358, Sill Optics GmbH, Wendelstein, Germany) at normal incidence. A laser beam operating at a repetition frequency of 100 kHz was guided by a galvanometer scanner head (SUPERSCAN IV-30, Raylase GmbH, Weßling, Germany) to directly write lines on the surface of a ZnO substrate at a constant speed of 5000 mm/s. Thus, one-pulse irradiated spots arranged in a line with a spacing of 50 μm can be easily obtained by a single scan (Figure 2a). Pulses with energies ranging from 6 μJ to 60 μJ were used to obtain the one-pulse irradiated spots; as shown in Figure 2b, multi-pulse irradiated samples were obtained by irradiating the laser pulses (pulse energy: 30 μJ) upon the same spot, and the pulse numbers (*N*_pulse_: 1–11) were controlled by adjusting the irradiating time and repetition rate (0.01–100 kHz). After laser irradiation, the samples were separately immersed in acetone and purified water for three minutes of ultrasonic cleaning. The irradiated areas were observed by a scanning electron microscope (SEM, TM4000pulseI, Hitachi, Japan, at 5 kV) and a confocal laser scanning microscope (CLSM, LEXT ILS4100, Olympus, Tokyo, Japan) to attain the morphological details (radius, cleavage depth). The cathodoluminescence (CL, MonoCL4, Gatan Inc., Pleasanton, CA, USA with JSM-7800F, JEOL, Tokyo, Japan) of the laser-irradiated spots (repetition rate, 10 Hz) was also measured to analyze their luminescence properties and get their surface defect information.

## 3. Results and Discussion

**Figure 3:**
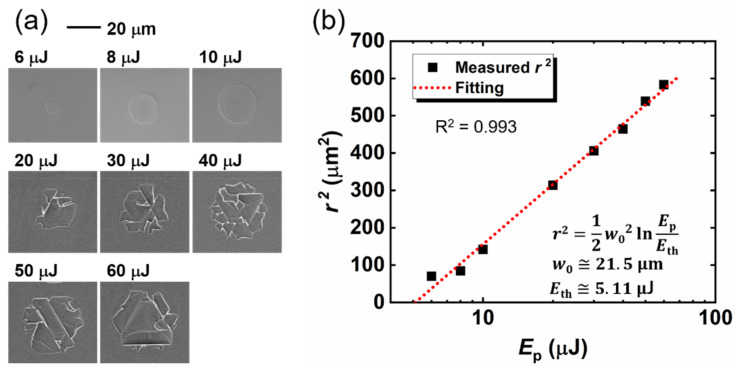
(**a**) SEM image of one-pulse irradiated spots. (**b**) The square of the spot radius (black squares) is plotted as a function of the pulse energy. The slope of the linear fitting yields the beam radius at the surface (*w*_0_). The extrapolation to zero shows the ablation threshold in the energy (*E_th_*) of one-pulse irradiation.

Figure 3a shows the SEM images of the one-pulse irradiated spots on the ZnO substrate surface. Broken hexagonal cleavages are observed when *E_p_* > 10 μJ. The laser-induced cleavages are centered at the irradiated spot and surrounded by a no-cleavage zone. This central location of cleavage is thought to be caused by the Gaussian energy distribution of the laser beam, whose center has the highest intensity. The Gaussian laser beam propagation in the *z*-direction can be given by the following:

(1) $$F\left(x,\;y\right)=F_{0}e^{-{\left(\frac{1}{w_{0}}\right)}^{2}2\left[{\left(x-x_{0}\right)}^{2}+{\left(y-y_{0}\right)}^{2}\right]},$$

where *F* is the laser fluence (energy per unit area) and *F*_0_ is its maximum value, *w*_0_ is the radius at which the fluence falls to *F*_0_/*e*^2^, and (*x*_0_, *y*_0_) is the beam center of the profile [23,24,25,26]. The index (*x* − *x*_0_)^2^ + (*y* − *y*_0_)^2^ denotes the square of the distance to the center, i.e., the square of the radius (*r*) from the aerial view. Substituting *r*^2^ = (*x* − *x*_0_)^2^+(*y* − *y*_0_)^2^ into Equation (1) and replacing *F*_0_ with 2*E*_p_/($\pi w_{0}^{2}$), we obtain the following:

(2) $$r^{2}=\frac{1}{2}w_{0}^{2}\ln\frac{E_{p}}{E_{th}},$$

where *E_p_* is its pulse energy, and *E_th_* is the ablation threshold energy. Based on our SEM images, we measured the ablation areas of the one-pulse irradiated spots (shown by black squares in Figure 3b) and obtained the *r*^2^ value. The red broken line in Figure 3b shows the fitting curve described by Equation (2); according to the simulated values of *E_th_* (5.11 μJ) and *w*_0_ (21.5 μm), we can obtain the ablation threshold, *F_th_* = 0.35 J/cm^2^.

**Figure 4:**
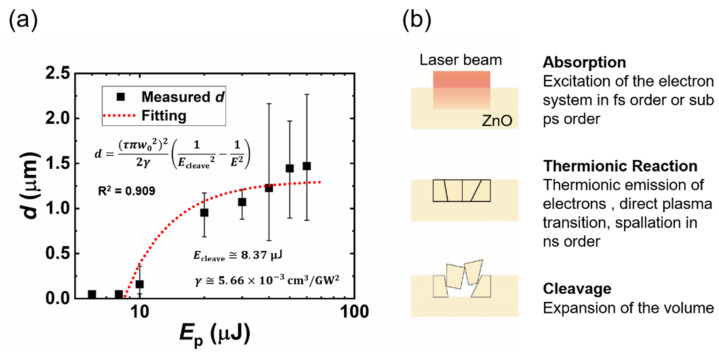
(**a**) The cleavage depth (*d*) (black squares) is plotted as a function of the pulse energy. The red broken line shows a fitting result based on a three-photon absorption model. The extrapolation to zero shows the laser-induced cleavage threshold energy (*E_cleave_*). (**b**) Image of the laser-induced surface-cleavage model.

The bandgap of ZnO (3.3 eV) is as wide as three times that of Si (1.1 eV); when a laser pulse with a wavelength of 1030 nm (*E*_photon_ = 1.2 eV) is irradiated to the Si substrate, most energy will be absorbed by the surface. On the contrary, in the case of ZnO, multi-photon absorption will predominate, and some of the energy will propagate into the ZnO substrate. The cleavage depths (*d*) of the one-pulse irradiated spots are plotted as a function of the pulse energy, as shown in Figure 4a. For the one-pulse irradiated samples in our work, the pulse energy irradiated to the ZnO substrate is below 60 μJ, and the laser-induced cleavage depth is less than 3 μm according to the CLSM measurement; this suggests that the propagation time from the surface to the cleaved valley of the laser pulse is shorter than 20 fs (*t* = *nd*/*c*, where *t* is the propagation time, *n* is the refractive index of ZnO, and *c* is the speed of light). Hence, the multi-photon absorption (femtosecond or sub-picosecond order from the laser–matter interaction) will be finished before the thermionic reaction (nanosecond order from the laser–matter interaction); the expansion caused by the thermionic reaction then leads to cleavage [27,28]. Figure 4b presents an image of the laser-induced surface cleavage. The coefficients of the two-photon absorption (2PA) and three-photon absorption (3PA) of the bulk ZnO were measured by Vivas et al. [29] and He et al. [30], respectively. Vivas reported that a saturable one-photon absorption (1PA) was observed due to the deep levels; a mixture of 1PA, 2PA, and 3PA was observed for the excitation from 530 nm to 800 nm and, when the ZnO was excited by light with a wavelength from 820 nm to 980 nm, 3PA predominated. Because the 1030-nm wavelength is close to the 3PA-predominant region in the result given by ViVas, we propose a 3PA-based laser-induced cleavage model. When a laser beam with an average intensity of *I* propagates through the sample along the z-direction, the 3PA result can be written as follows:

(3) $$\frac{dI}{dz}=-\gamma I^{3};$$

this equation can be solved to obtain the following:

(4) $$I\left(z\right)={\left(2\gamma z+\frac{1}{I_{0}^{2}}\right)}^{-\frac{1}{2}},$$

where *γ* is the 3PA coefficient, *I*_0_ is the irradiance of the laser pulse (power per unit area, *E_p_*/($\tau \pi w_{0}^{2}$)) [31,32,33], and *I*(*z*) is the laser intensity at position *z* of laser propagation direction inside the ZnO substrate. Equation (4) can also be rewritten as follows:

(5) $$d=\frac{{\left(\tau \pi w_{0}^{2}\right)}^{2}}{2\gamma }\left(\frac{1}{E_{cleave}{}^{2}}-\frac{1}{E_{p}^{2}}\right),$$

where *d* is the cleavage depth, *τ* is the pulse duration (700 fs), *w*_0_ is the focused radius (21.5 μm, simulated by Figure 2b), *E_cleave_* is the threshold energy of laser-induced cleavage for our processing system, and *E_p_* is the energy of incident laser pulse. The measured cleavage depth was fitted based on Equation (5) and shown in Figure 4a. The 6-μJ irradiated spot was neglected for the fitting because the intensity may be too low to encourage multi-photon absorption [34]. The fitting curve agrees well with the measured results, and we obtained a simulated value of *E_cleave_* (8.37 μJ) indicating the minimum pulse energy for laser-induced cleavage. The simulated γ in our work is 5.66 cm^3^/GM^2^, which agrees with the result measured by He et al. The thermal properties also contribute considerably to the cleavage process; ZnO has a higher thermal-expansion coefficient (α_⊥_~5 × 10^−6^ K^−1^, α//~3 × 10^−6^ K^−1^ [35,36,37] and a much lower thermal conductivity (κ < 50 W·m^−1^·K^−1^ [37,38,39,40]) than Si (α~2.6 × 10^−6^ K^−1^ [41,42,43], κ~150 W·m^−1^·K^−1^ [41,44]) at a temperature of ~300 K. These thermal properties indicate that a larger expansion in a smaller volume will be induced by laser irradiation in the ZnO substrate than in Si.

**Figure 5:**
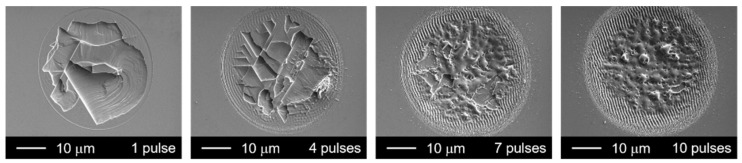
ZnO surface irradiated by laser pulses with the same pulse energy of 30 μJ but different pulse numbers (repetition rate: 10 Hz).

**Figure 6:**
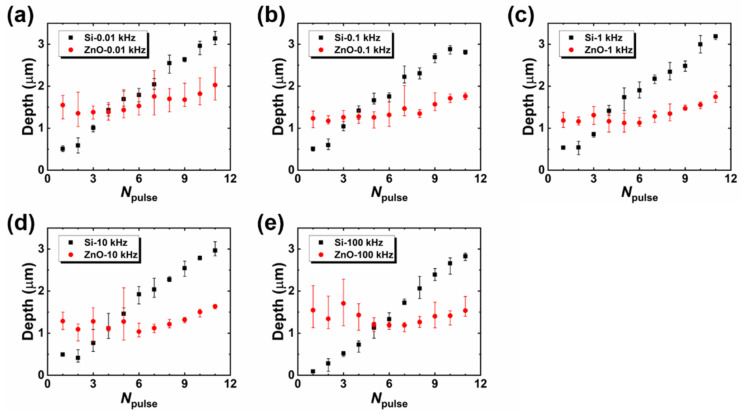
Depths of the multi-pulse irradiated spots of ZnO (red squares) and Si (black squares) under different repetition rates in the range 0.01–100 kHz (**a**–**e**).

Although the one-pulse irradiated spots showed cleavage marks if the pulse energy was higher than the threshold energy, the laser-induced cleavage did not remain when the same spot was irradiated by multiple pulses. As shown in Figure 5, when the pulse number reaches four, the conspicuous melt is deposited on the no-cleavage zone, and the melt area increases with pulse number. Consequently, the 10-pulse irradiated spot becomes a fully melted surface. This cleavage–melt shift was found for all of the samples under different repetition rates according to their CLSM measurements, and it may be caused by the vestigial heat from the initial laser–matter interaction or the change in absorption related to the surface defect arising from previous irradiations. As shown in Figure 6, the depth of the laser-irradiated Si increases almost linearly with the number of pulses. This linear increase indicates that there is no mechanistic change in the ablation process as the pulse number increases. The melting process consistently accompanies the increase in laser pulses. On the other hand, the depth of the laser-irradiated ZnO seems to be randomly distributed for pulse numbers less than five, and from the fifth pulse, the depth linearly increases with the laser-pulse number. The cleavage–melt shift occurred for all samples with laser repetition rates from 10 Hz to 100 kHz. However, the repetition rate is associated with the waiting time for the next pulse, which will vary the cooling time for the irradiated spot between pulses. Hence, when the repetition rate is low enough, the longer cooling time is possible to change the formation process of the surface defect. Therefore, the change in repletion rate may not affect whether the melt–cleavage shift happens, but it is possible to shift the *N*_pulse_ where the melt-cleavage shift was induced. 

**Figure 7:**
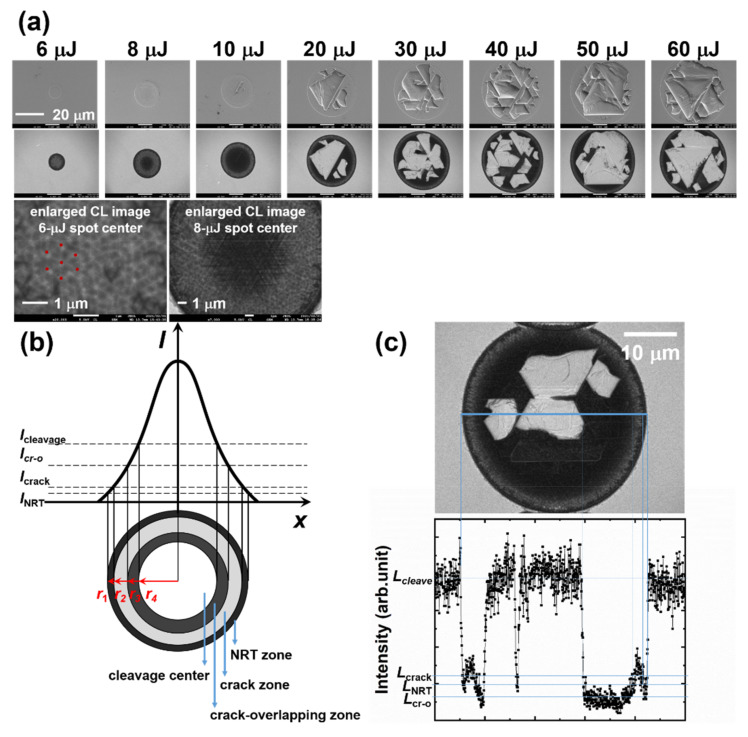
(**a**) SEM (top) and CL (bottom) images of the one-pulse irradiated spot. The red points in the enlarged CL image of the 6-μJ irradiated spot are the vertices of the triangles that combine to form the hexagon. The enlarged CL image of the 8-μJ irradiated spot shows a crack-overlapping center surrounded by the crack zone. (**b**) Image of the cleavage center, crack zone, crack-overlapping zone, and NRT zone of the laser-irradiated spot when the intensity is sufficiently high to induce surface cleavage. (**c**) The CL image and a line profile of luminescence intensity in a 20-μJ irradiated spot.

Figure 7a shows the SEM and CL images of the one-pulse irradiated spots with different pulse energies. In the enlarged CL image of the 6-μJ irradiated sample, nano-cracks with triangular or hexagonal (six triangular pieces) shapes were observed at the center of the spot. In these nano-cracks, luminescence was detected from the edges, but the centers were relatively dusky. Moreover, 6 μJ is a relatively low pulse energy close to the processing threshold (5.66 μJ), which may not be high enough to induce nano-cracks inside the ZnO substrate; this suggests that the luminescence should be generated from the exposed ZnO and escape from the shallow cracks. A dark center surrounded by a round area with higher luminescence intensity (a donut shape) was observed in the CL image of the 8-μJ irradiated spot center, and the enlarged CL image shows that the overlapping cracks constituted the dark center. In a Gaussian beam, the center has the highest intensity and this gradually decreases toward the edge. The 8-μJ irradiated center has a higher intensity level than that of the 6-μJ irradiated spot (crack zone), leading to cracks under the surface that result in a crack-overlapping zone. The overlap of cracks can obstruct the escape of luminescence from the inside, and constitutes the dark center. Moving outwards from the center of the 8-μJ irradiated spot, the intensity will decrease to the same level of the 6-μJ irradiated center, such that a luminous donut with the pattern of triangles and hexagons as seen in the center of the 6-μJ irradiated spot is observed surrounding the dark center. Both the 6- and 8-μJ irradiated spots have dark rings in their outermost areas, which have the lowest intensity in a laser spot. In this area, the intensity may be too low to induce nano-cracking. Non-radiative defects may arise due to this low-intensity irradiation (as shown in Figure 7b) and give rise to the dark ring (NRT zone). When the pulse energy is higher than 10 μJ, the area of the laser-induced cleavages was observed to increase with the pulse energy, and the exposed surface shows a relatively strong luminescence compared to the surrounding area.

**Figure 8:**
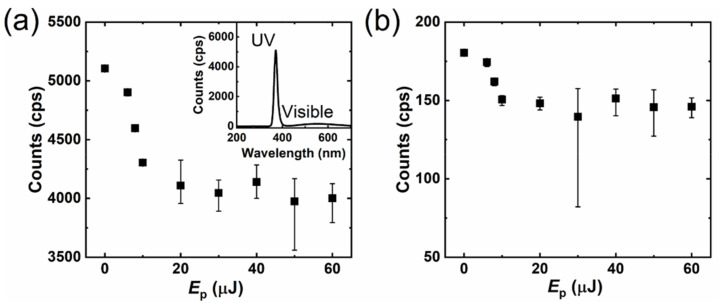
The intensities of the UV (**a**) and green-light (**b**) luminescence of the one-pulse irradiated spots. The error bar using minimum and maximum was obtained from five samples. The inserted graph shows the CL spectrum of the unprocessed ZnO surface whose index is 0 J in graphs (**a**,**b**).

The luminescence properties of ZnO have been reported in detail on numerous occasions [45,46,47,48,49,50,51,52,53,54]. The consensus is that UV luminescence should be attributed to the near-band emission, but the visible luminescence associated with various intrinsic defects can show various wavelengths. Figure 8 shows the intensity of the UV (a) and visible (b) peaks of the one-pulse irradiated samples according to their CL spectra. For the one-pulse irradiated samples, UV emission appeared at 371.5 ± 0.3 nm with a full width at half maximum (FWHM) of 16.3 ± 0.2 nm, while the green peak appeared at 549.3 ± 8.0 nm with an FWHM of 179.2 ± 2.8 nm. For the bulk surface, UV emission appeared at 371.6 ± 0.3 nm with an FWHM of 16.3 ± 0.0 nm, while the green peak appeared at 556.4 ± 5.6 nm with an FWHM of 177.5 ± 1.8 nm. The green luminescence in our ZnO samples has a similar wavelength to the reports by Zhang et al. [43] and Camarda et al. [53]; it is related to the defects of the ionized oxygen vacancies. For both UV and green luminescence, a decrease process was observed when *E*_p_ < 20 μJ; for higher pulse energies, the intensity randomly changed with the pulse energy. When *E*_p_ ≥ 20 μJ, the intensity changed randomly, but the average intensity remained at a relatively constant level, which was decreased dramatically from the bare ZnO substrate (*E*_p_ = 0 J) to *E*_p_ = 20 μJ.

When the intensity was high enough to induce cleavage, the luminescence intensity should be related to the areas of the cleavage center (*S*_cleavage_), the crack-overlapping zone (*S*_cr-o_), the crack zone (*S*_crack_), and the NRT zone (*S*_NRT_). Figure 7c shows a CL image and a line profile of the luminescence intensity for a 20-μJ irradiated spot. The exposed surface has the same luminescence intensity as the unprocessed surface (*L*_surface_ = *L*_cleave_), which is higher than those of the crack-overlapping, crack, or NRT zones (*L*_surface_ = *L*_cleave_ > *L*_crack_ > *L*_NRT_ > *L*_cr-o_). Because *L*_surface_ =*L*_cleave_, the detected CL intensity of a cleavage spot is related to *S*_cr-o_, *S*_crack_, and *S*_NRT_.

As shown in Figure 7b, for an ideal laser-induced cleavage surface, the intensity level for cleavage (*I*_cleavage_) can be written as follows:

(6) $$I_{\mathrm{cleavage}}=I_{0}e^{-2{\left(\frac{r_{4}}{w_{0}}\right)}^{2}},$$

and that for inducing the crack-overlapping zone (*I*_cr−o_) can be written as follows:

(7) $$I_{\mathrm{cr}-o}=I_{0}e^{-2{\left(\frac{r_{3}}{w_{0}}\right)}^{2}}.$$

According to Equations (6) and (7), we can obtain the following:

(8) $$S_{\mathrm{cr}-o}=\pi (r_{3}^{2}-r_{4}^{2})=\frac{\pi w_{0}^{2}}{2}\ln\frac{I_{\mathrm{cleavage}}}{I_{\mathrm{cr}-o}},$$

where *I*_cleavage_ is the intensity for inducing the cleavage, and *I*_cr−o_ is the intensity for inducing the crack-overlapping zone. Here, *I*_cleavage_ and *I*_cr-o_ should be a constant for a settled processing system and a specified material, indicating that the crack-overlapping zone should have an immutable area *S*_cr-o_ in an ideally cleaved spot. In the same way, *S*_crack_ and *S*_NRT_ can also be obtained as $\frac{\pi w_{0}^{2}}{2}\ln\frac{I_{\mathrm{cr}-o}}{I_{\mathrm{crack}}}$ and $\frac{\pi w_{0}^{2}}{2}\ln\frac{I_{\mathrm{crack}}}{I_{\mathrm{NRT}}}$, respectively, and independent to the energy of the incident laser pulse. The immutability of such areas suggests that an ideally cleaved surface should have a constant intensity of CL luminescence.

Due to the formation of the NRT, crack, and crack-overlapping zone, the UV and green luminescence decrease until the pulse energy increases to the level at which surface cleavage is induced. When the pulse intensity is high enough to induce surface cleavage, the crack-overlapping area, crack, and NRT zones will be constant. The surface exposed by cleavage increases with the pulse energy, but the luminescence intensity of the exposed surface is almost the same as that of the unprocessed surface. Consequently, the luminescence intensity will be constant for an ideal laser-induced cleavage surface.

**Figure 9:**
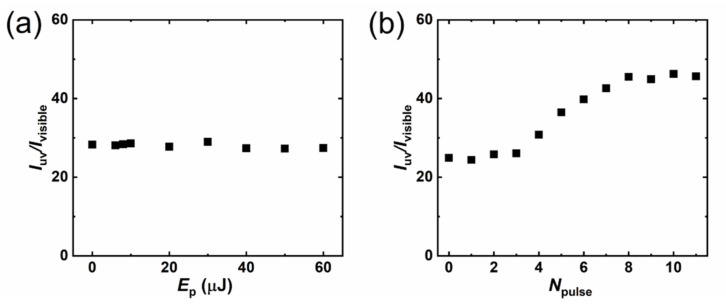
The intensity ratio of UV and green luminescence. (**a**) One-pulse irradiated spot; (**b**) multi-pulse irradiated spot (repetition rate, 10 Hz). The zero-J and zero-pulse samples in the graph indicate the results of unprocessed ZnO.

**Figure 10:**
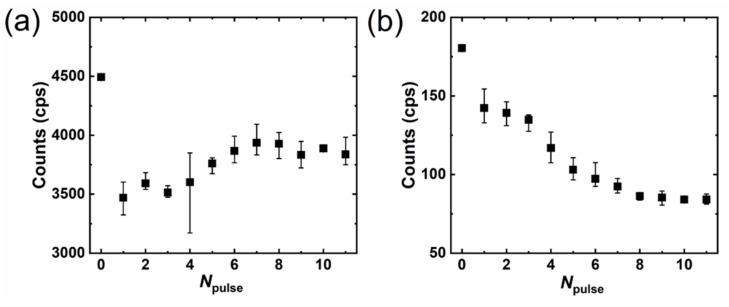
The intensities of UV (**a**) and green (**b**) luminescence for multi-pulse irradiated spots (repetition rate, 10 Hz). The error bars using minimum and maximum are obtained from four samples. The zero-pulse sample in the graph indicates the result for unprocessed ZnO.

Figure 9 shows the UV–green ratio of luminescence intensity; for one-pulse irradiated samples (Figure 9a), this ratio showed a smooth distribution with almost no change as the pulse energy increased. On the other hand, the ratio of multi-pulse irradiated samples (Figure 9b) started to increase after four pulses and saturated at eight. This suggests that the surface-defect state is not overly affected by the pulse energy in the one-pulse irradiated samples, but can be changed by multi-pulse irradiation. Figure 10 shows the UV (a) and green (b) peak intensities of CL luminescence for the multi-pulse irradiated samples. For these samples, UV emission appeared at 372.2 ± 0.4 nm with an FWHM of 17.0 ± 0.6 nm, while the green peak appeared at 543.1 ± 4.9 nm with an FWHM of 172.3 ± 3.3 nm. The UV intensity dramatically decreased for the first pulses due to a sudden increase in the crack-overlapping, crack, and NRT zones. The UV intensity randomly changed from one to three pulses, continued increasing from four to seven pulses, and saturated at eight pulses. On the other hand, the intensity of the green peak kept decreasing until saturating at eight pulses. This result indicates that the defects contributing to green luminescence decreased with the pulse number. Because the similar depth change via *N*_pulse_ was obtained for the samples irradiated with a repetition rate from 10 Hz to 100 kHz (Figure 6), an increasing and then saturating process of *I*_uv_/*I*_visible_ (Figure 9b) can also be predicted for the samples irradiated under repetition rates from 100 Hz to 100 kHz.

**Figure 11:**
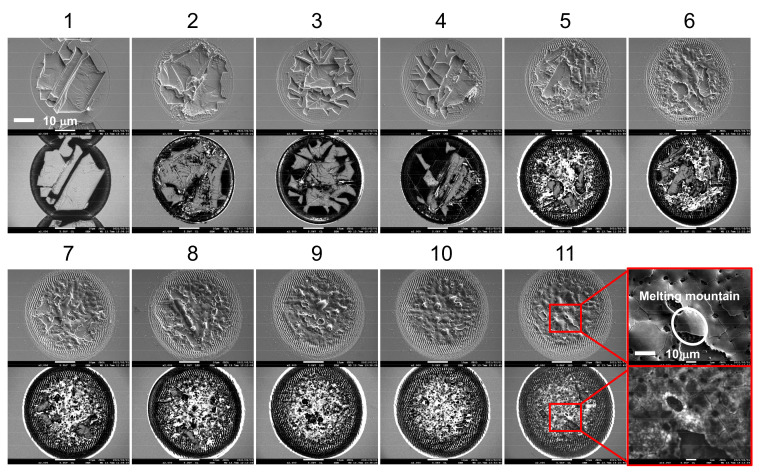
SEM (top) and CL (bottom) images of multi-pulse irradiated samples. The numbers above the SEM images indicate the number of pulses irradiated upon each sample.

As shown in Figure 11, before the fourth pulse, laser-induced cleavage is the main phenomena of laser processing and the randomly exposed surface leads to a variable UV intensity. Meanwhile, the melted material deposited on top of the crack-overlapping, crack, and NRT zones slightly increased with the pulse number. Compared to the bulk surfaces of ZnO, these areas should have a higher absorptivity of laser pulses, causing the melt to be preferentially deposited on these areas. The melted material also showed a relatively high luminescence intensity. After five-pulse irradiation, the melt increased quickly with the pulse number, and when eight pulses were irradiated onto the spot, a fully melted surface was formed. As shown in the enlarged SEM and CL images of the 11-pulse irradiated sample, the melting mountain has a smooth surface in the SEM image; however, the CL images suggest that the melting mountain is formed by many nano phosphors induced by laser ablation. In the high-temperature, high-pressure center of the irradiated spot, the ablated pieces were heated and melted together. Consequently, the green luminescence in our work is thought to be caused by transitions from oxygen-vacancy (V_o_) defects to the valance band, as V_o_ decreased with pulse number under laser irradiation in oxygen-rich conditions [48,49,51,52,53]. After eight pulses, the fully melted surface was formed and the melted area remained almost changeless while the pulse number increased, leading to saturation of the CL luminescence. The lowest *I*_UV_/*I*_visible_ in Figure 9b, and the highest intensity of green luminescence for the bare ZnO surface (*N*_pulse_ = 0), suggest that a bare surface with suitable V_o_ density may be easier to induce the MPA and the surface cleavage when it is irradiated by ultrafast laser pulses.

## 4. Conclusions

In summary, laser-induced cleavage on the bulk ZnO surface was observed under single-pulse irradiation (wavelength: 1030 nm, pulse duration: 700 fs). A three-step process of multi-photon absorption, expansion, and cleavage was proposed for the laser-induced cleavage of ZnO. When ZnO is irradiated by laser pulses with a 1030-nm wavelength, the 3PA due to the wide bandgap will be predominate and results in surface cleavages. This 3PA-induced surface cleavage differs from the general melt process caused by 1PA that occurred on the surface of Si or other metals. The pulse energy-dependent cleavage depth was quantitatively analyzed by a three-photon absorption model, and the three-photon absorption coefficient was calculated to be 5.66 cm^3^/GW^2^. The cleavage–melt shift of laser processing on the surface of the ZnO substrate was observed and characteristically analyzed in terms of the change in the surface-defect states during multi-pulse irradiation. The laser-induced cleavage process will be caused by 3PA of the first pulse. Then, a mixed process of cleavage and melt occurs from two to four pulses, resulting in the formation of the crack-overlapping, crack, and NRT zones, with absorptions higher than that of the unprocessed surface; after four pulses, the general laser-induced melt process comes to dominate. Moreover, the intrinsic defect of V_o_ decreased with the input of the pulse number, which could decrease the intensity of green luminescence and increase the UV–visible ratio of luminescence intensity. Additionally, surface cleavage could be possibly induced on other wide-bandgap materials by MPA under ultrafast laser irradiations, for example, gallium nitride, which also has cleavability and a wide bandgap of 3.4 eV. These results can potentially be used to extend femtosecond laser-based surface-modification processing methods, or to design laser-induced functional surfaces of ZnO or other wide-bandgap materials with a wide bandgap and suitable thermal properties (i.e., a high thermal expansion coefficient and low thermal conductivity).

## Data Availability

The data that support the findings of this study are available from the corresponding author upon reasonable request.
